# Do riparian forest strips in modified forest landscapes aid in conserving bat diversity?

**DOI:** 10.1002/ece3.5048

**Published:** 2019-03-27

**Authors:** Farah Carrasco‐Rueda, Bette A. Loiselle

**Affiliations:** ^1^ School of Natural Resources and Environment University of Florida Gainesville Florida; ^2^ Department of Wildlife Ecology and Conservation University of Florida Gainesville Florida; ^3^ Center for Latin American Studies University of Florida Gainesville Florida

**Keywords:** agriculture, Chiroptera, conservation, Peru, Phyllostomidae

## Abstract

Agricultural practices lead to losses of natural resources and biodiversity. Maintaining forests alongside streams (riparian forest strips) has been used as a mechanism to minimize the impact of clearing for agriculture on biodiversity. To test the contribution of riparian forest strips to conserve biodiversity in production landscapes, we selected bats as a biodiversity model system and examined two dimensions of diversity: taxonomic and functional. We compared bat diversity and composition in forest, with and without stream habitat, and in narrow forest riparian strips surrounded by areas cleared for agriculture. We tested the hypothesis that riparian forest strips provide potential conservation value by providing habitat and serving as movement corridors for forest bat species. Riparian forest strips maintained 75% of the bat species registered in forested habitats. We found assemblage in sites with riparian forest strips were dominated by a few species with high abundance and included several species with low abundance. Bat species assemblage was more similar between sites with streams than between those sites to forests without stream habitat. These results highlight the importance of stream habitat in predicting presence of bat species. We registered similar number of guilds between forest sites and riparian forest strips sites. Relative to matrix habitats, stream and edge habitats in riparian forest strips sites were functionally more diverse, supporting our hypothesis about the potential conservation value of riparian forest strips. Results from this study suggest that maintaining riparian forest strips within cleared areas for agricultural areas helps conserve the taxonomic and functional diversity of bats. Also, it provides basic data to evaluate the efficacy of maintaining these landscape features for mitigating impacts of agricultural development on biodiversity. However, we caution that riparian forest strips alone are not sufficient for biodiversity maintenance; their value depends on maintenance of larger forest areas in their vicinity.

## 1 INTRODUCTION

Agricultural practices lead to losses of natural resources and biodiversity (Godfray & Garnett, 2014). One mechanism to minimize the impact of clearing for agriculture and other production activities on biodiversity is to maintain riparian forest strips (insects: Barlow et al., 2010, Brito et al., 2017, Gray, Lewis, Chung, & Fayle, 2015, Gray, Slade, Mann, & Lewis, 2014; macroinvertebrates: McClain, 2002; fish: Giam et al., 2015; birds: Machtans, Villard, & Hannon, 1996, Mitchell et al., 2018, Whitaker & Montevecchi, 1999; bats: Lloyd, Law, & Goldingay, 2006, Mullin, 2015; small mammals: Al‐Khudhairy Gutierrez, 2015; Chapman & Ribic, 2002; Cockle & Richardson, 2003; Darveau, Labbe, Beauchesne, Belanger, & Huot, 2001; large and medium‐sized mammals: Paolino et al., 2018, Phoebus, Segelbacher, & Stenhouse, 2017, Zimbres, Peres, & Machado, 2017). These strips may become prominent features in agricultural landscapes and may assume disproportionate roles in protecting biodiversity outside protected areas (Arriaga‐Flores, Castro‐Arellano, Moreno‐Valdez, & Correa‐Sandoval, 2012; Mendenhall, Karp, Meyer, Hadly, & Daily, 2014; Naiman, Decamps, & Pollock, 1993). Within agricultural landscapes, riparian forest strips may help conserve water resources, improve water quality, harbor animals that serve as bio‐control agents, provide connections between forest fragments, and act as physical barriers to destructive fires, radiation fluxes, winds, and pests (Muscutt, Harris, Bailey, & Davies, 1993; Saunders, Hobbs, & Margules, 1991; Zanuncio, Mezzomo, Guedes, & Oliveira, 1998). In recognition of these benefits, the maintenance of riparian forest strips is supported by legislation in several tropical countries (Barlow et al., 2010; McClain & Cossio, 2003; Pereira et al., 2018) and, in the case of Peru, by Law 29,338 “Hydric Resources Law.” However, the efficacy of riparian forest strips in maintaining biodiversity in cleared agricultural landscapes has been primarily examined in temperate areas (Chapman & Ribic, 2002; Cockle & Richardson, 2003; Darveau et al., 2001; Hagar, 1999; Machtans et al., 1996; but see de la Pena‐Cuellar, Benitez‐Malvido, Avila‐Cabadilla, Martinez‐Ramos, & Estrada, 2015; Lees & Peres, 2008; Lourenco, Gomes, Pinheiro, Patricio, & Famadas, 2014; Mitchell et al., 2018; Paolino et al., 2018). Given the biodiversity richness of tropical forests, especially lowland wet forests, and the rapid expansion of large‐scale agriculture in these areas, we need additional work to demonstrate the conservation value, if any, of riparian forest strips in tropical regions. Our study was motivated by understanding the importance of riparian forest strips as a mitigation strategy to help maintain bat taxonomic and functional diversity in forested areas undergoing agricultural development.

Among agricultural activities, development of palm plantations has expanded rapidly throughout the world's lowland tropics, resulting in considerable loss of forest (Curtis, Slay, Harris, Tyukavina, & Hansen, 2018; Phalan et al., 2013). Much of this development is focused on African oil palm (*Elaeis guianensis*), which has raised considerable concern because of its negative impacts on biodiversity (Vijay, Reid, Finer, Jenkins, & Pimm, 2018; Wilcove & Koh, 2010). Development of oil palm has exploded in the last decade concentrated in Asia and Africa, and only more recently has begun to play a significant role in South America (Pirker, Mosnier, Kraxner, Havlik, & Obersteiner, 2016). Peru is a good example where oil palm plantations, which covered only a small extent a few years ago, are now expanding rapidly and acting as an important driver of deforestation in the Amazon region. Although not as widespread as oil palm plantations, peach palm plantations, which are the development focus for this study, are expected to have similar negative consequences as forests are cleared to make way for palm plants.

We focus our study on bats as a biodiversity model system because they serve as good indicators of ecosystem health due to their species diversity, have varied life histories and morphologies, and are important in providing a variety of ecological services (Bennett, Radford, & Haslem, 2006; Castro‐Luna, Sosa, & Castillo‐Campos, 2007; Fenton & Rautenbach, 1998; Hein, Castleberry, & Miller, 2009). Bats play key roles in forest regeneration and ecosystem dynamics through the ecological services they provide combined with their high mobility and use of a variety of habitats (Gorchov et al., 2013; Kasso & Balakrishnan, 2013).

Previous work has highlighted the impact of habitat loss and land‐use change on bat diversity (Albrecht, Meyer, & Kalko, 2007; Garcia‐Morales, Badano, & Moreno, 2013; Henry, Cosson, & Pons, 2007; Kalko, 1997; Klingbeil & Willig, 2009; Loayza & Loiselle, 2008; Willig, 1986). Vulnerability to forest disturbance is not random with certain species (e.g., diet and roost specialists, species with small home ranges) being differentially impacted. Nonrandom species loss or decline likely results in changes in species diversity and composition, as well as functional diversity, which is particularly relevant for ecological function, and links species to higher order ecological and ecosystem processes (Petchey & Gaston, 2006). Changes in forest cover may also impact bat behavior and thus, alter the ecological services they provide (Klingbeil & Willig, 2009; Mello et al., 2011; Westcott, Bentrupperbaumer, Bradford, & McKeown, 2005). For example, some bats avoid crossing open areas presumably because of the risk of predation by raptors and other aerial predators (Chacon‐Madrigal & Barrantes, 2004). This avoidance of open areas may impact pollinator or seed dispersal services by affecting pollen and seed movement in fragmented landscapes. The presence of forest corridors, such as riparian forest strips, in modified landscapes has long been suggested as a mechanism to mitigate deforestation impacts (Bennett & Zurcher, 2013; Estrada & Coates‐Estrada, 2001; Hein et al., 2009; MacDonald, Tattersall, Service, Firbank, & Feber, 2007; Meyer, Struebig, & Willig, 2016). The degree to which these corridors are effective in maintaining taxonomic and functional diversity in the landscape may depend on which forest species use or rely on them as corridors or foraging habitat (de la Pena‐Cuellar et al., 2015; Galindo‐Gonzalez & Sosa, 2003; Lourenco et al., 2014; Meyer et al., 2016).

Our hypothesis is that riparian forest strips provide potential conservation value by providing habitat and serving as movement corridors for forest bat species. If bats use riparian forest strips as foraging habitat, then we predict that the species composition of bats should more closely resemble that found alongside forest stream habitats. If bats use riparian forest strips as movement corridors in agricultural landscapes, then we predict that habitat may not emerge as factor that explains variation in species diversity of composition among sites. If bats use riparian forest strips as both movement corridors and foraging habitat, then we predict that measures of bat diversity may be similar to other forest sites, or perhaps even exceed forest sites without stream habitats. Our examination of bat diversity included two dimensions: taxonomic diversity comprising species richness, abundance, assemblage composition; and functional diversity as defined by guilds (Kalko, 1997; Schnitzler & Kalko, 1998).

Testing the effectiveness or failure of agricultural models for conserving biodiversity is key in promoting their implementation or improvement, and in justifying the costs incurred, in this case, for maintaining the riparian forest strips in the agricultural landscape. Our goal is to address this need by taking advantage of the establishment of a new peach palm (*Bactris gasipaes*) plantation, which is maintaining forest cover alongside streams (i.e., riparian forest strips) in accordance with Peruvian legislation.

## 2 MATERIALS AND METHODS

### 2.1 Study location

The San Martín Region in north‐central Peru is one of the country's hotspots of deforestation (Finer, Novoa, & Garcia, 2018), but also is one of the regions in the country that has ecological and economic zoning map. This study was conducted on a private property within the Amazon plain of north‐central Peru (San Martín Department, Lamas Province, Caynarachi District, UTM 18 S 352,520 E, 9,316,392 N, 190 m.a.s.l.), inside the buffer zone of Cordillera Escalera Regional Protected Area. The principal vegetation types in the private property were old (i.e., 15–20 years since abandonment of agriculture; ~40%) and recent (<10 years old) secondary forest (~20%), *Mauritia flexuosa* palm swamps (5%), pasture (30%), and croplands (5%). In a 1,350 ha area zoned for development of a plantation of *Bactris gasipaes* palms, 15–25 m strips of natural forest vegetation were protected along each side of all permanent streams (typically 2–4 m wide) as required by Peruvian law (Law 29,338 “Hydric Resources Law” – Autoridad Nacional del Agua). Fieldwork on bats occurred during the 2014 dry season (May‐July). The field team was led by FCR and included two local guides and, intermittently, a volunteer.

### 2.2 Sampling design

**Figure 1:**
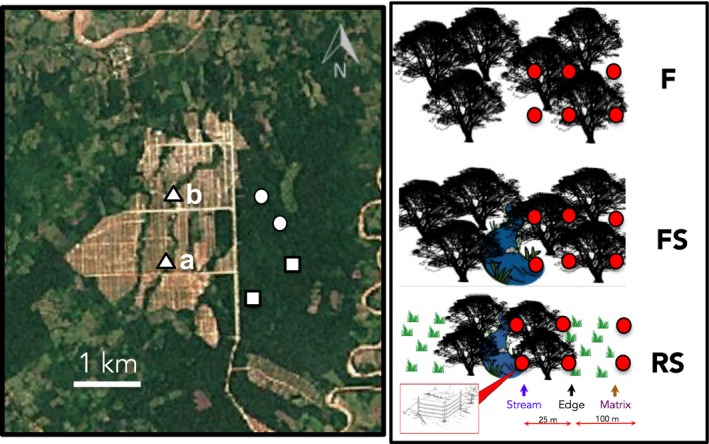
Sampling design: Location of study sites (left) shown on a satellite image as background (Source: Google Earth, December 30, 2015. Downloaded on January 11, 2018). Squares on the satellite image represent forest treatment (F) sites, circles represent forest with stream treatment (FS) sites, and triangles represent riparian forest strips in recent clearings treatment (RS) sites. Site “a” was cleared <1 month prior and site “b” was cleared >2 months prior. On the right panel, we depict the arrangement of mist‐nets within each treatment, where red dots represent a pair of mist‐nets in an “L” shape

After the forest clearing process but prior to planting of the palms, we established experimental units, hereafter sampling sites, in two forest sites without streams (F), two forest sites with streams (FS), and two sites where riparian forest strips (which included stream habitat and surrounding ~25 m wide forest buffer up to the edge) were surrounded by open areas where forest was clearcut (RS). One RS site was recently cleared less than a month prior to the first visit, and the other was cleared about two months before, although some adjacent land (~1 ha) had been cleared 5–12 years prior and maintained without forest cover (e.g., some small‐scale agriculture, pasture). All sites were separated by >400 m (Figure 1). We considered F, FS, and RS as treatments. We defined four habitat categories: “forest,” “stream,” “edge” (border of the forest adjacent to cleared areas), and “matrix” (i.e., open areas cleared of forest). Habitats were embedded within treatments. At F sites, only forest habitat was present; at FS sites, there were both stream and forest habitats; and three habitats—stream, edge, and matrix—were present at RS sites. In RS sites, stream and edge habitats formed part of the riparian forest strips. At all sites, the spatial and temporal structure of sampling was the same (see “*Bat sampling”*).

### 2.3 Bat sampling

We sampled 576 mist‐net hours (12 mist‐nets during 6 hr per night across 8 nights) per study site, where 1 mist‐net hour equals one mist‐net (12 m length by 3 m height; 36 mm mesh) opened for 1 hr. Mist‐nets were placed in “L” shaped pairs in each of the three “habitats” as shown in Figure 1. We used a total of four mist‐nets per habitat and 12 mist‐nets per site. At the two RS sites, four mist‐nets were placed along the stream, four “edge” mist‐nets were placed on the border of the riparian strip (~25 m from stream), and four “matrix” mist‐nets were placed 100 m away from the forest edge in the open area. At the two FS sites, four mist‐nets were placed in “stream” habitat and eight mist‐nets in “forest” habitat. At the two F sites, all 12 mist‐nets were located in “forest” habitat (Figure 1).

Each site was sampled during two visits (four nights each visit) separated by 14–30 days so as to minimize seasonal variation. At each visit, we opened the 12 mist‐nets at a site during four consecutive nights from 18:00 to 0:00 hr; nets were checked for bats every 30 min. For all individuals captured, we recorded body measurements, weight, sex, age, and reproductive status. Species were determined in the field using taxonomic keys (Aguirre, Vargas, & Solari, 2009; Diaz, Aguirre, & Basquez, 2011; Gardner, 2007). To enable identification of recaptures, prior to release we clipped a small section of hair on each bat's back (Harvey & Gonzalez Villalobos, 2007; Helbig‐Bonitz, Rutten, & Kalko, 2014; Klingbeil & Willig, 2009). Individuals that could not be identified were collected and maintained in 96% alcohol for later determination using museum resources. For bat taxonomic classification, we followed Gardner (2007) with modifications for species of the genus *Dermanura* (Vandenbussche, Baker, Wichman, & Hamilton, 1993). Specimens collected were deposited in the mammal collection of the Centro de Ecología y Biodiversidad (CEBIO), Lima, Peru (Supporting Information Table A1).

To test our hypothesis regarding the conservation value of riparian forest strips for maintenance of diversity, we included only bat species from the Phyllostomidae since mist‐nets are most effective sampling species from this family (Kalko, 1998). We evaluated the effectiveness of our sampling by plotting species accumulation curves based on the number of individuals captured. For this, we used function “specaccum” and the method “collector” from the R package “vegan” (Oksanen et al., 2016) in R environment (R Development Core Team, 2015).

### 2.4 Species richness

We estimated the expected richness of Phyllostomidae in each habitat by site using two nonparametric richness estimators: Chao 1 and Jackknife 1. We selected the Chao 1 index because of its ability to deal with uneven and small sampling sizes (Chao, 1984), and Jackknife 1 because it allows richness estimation bias reduction (Walther & Moore, 2005). We estimated both indices using the function “specpool” in the R package “vegan” (Oksanen et al., 2016).

We used individual‐based rarefaction curves to compare species richness at the treatment, individual site, and habitat level. At each level, we used subsamples with a size equal to the minimum number of individuals captured in a sample (treatments, sites, and habitats only for RS and FS sites since F sites only have forest habitat). In each case, we estimated mean expected species richness and 95% confidence intervals. Differences in species richness were considered to occur when confidence intervals between rarefaction curves did not overlap. Rarefaction curves from capture data were generated in R environment (R Development Core Team, 2015) using function “specaccum” from the R package “vegan” (Oksanen et al., 2016).

### 2.5 Species abundance distribution

We generated rank‐abundance distribution curves to visualize how diversity changes with treatment and habitat. We measured species relative abundance as capture rates (number of individuals captured by mist‐net hour) with 576 mist‐net hours accumulated at each site; 192 mist‐net hours in each habitat at RS sites, 192 mist‐net hours in stream habitat and 288 mist‐nets in forest habitat at FS sites, and 576 mist‐nets hours in forest habitat at F sites. Rank‐abundance curves were compared with Kolmogorov–Smirnov two‐sample tests. In addition, we compared rank‐abundance curves that corresponded to the habitats in the two RS sites to discard possible effect of time since clearing.

### 2.6 Assemblage composition

To estimate how well riparian forest strips maintain the bat assemblage, we compared species composition using capture data at treatment and habitat levels. For the first level, we pooled number of captures obtained in all mist‐nets across sites (six sites, FS1, FS2, F1, F2, RS1, and RS2). For the second level, we pooled data from mist‐net captures in each habitat by site. We generated nonmetric multidimensional scaling analysis (NMDS) to examine patterns in assemblage composition. First, we examined variation in the total number of captures (a) by species, and (b) by sampling location to determine whether standardization was required (coefficient of variation >50%). If needed, and to deal with super‐abundant and rarely captured species (the latter with <10 individuals, Medellin, Equihua, & Amin, 2000), we used Wisconsin double standardization (Bray & Curtis, 1957) that divides each element first by its column maximum and then by the row total. Finally, we created a Bray–Curtis dissimilarity matrix and used it to generate nonmetric multidimensional scaling analysis (NMDS) of bat composition by site. Low stress values indicate a good representation of the distance between objects in the *n*‐dimensional space. We reported which bat species had high and low positive loadings on both first and second axes to better understand what separates sites in terms of their bat species composition. We repeated these analyses for the habitat level. To test if there were differences among groups, we used permutational multivariate analysis of variance (PerMANOVA), a nonparametric analysis. In order to test whether similarity in bat composition was best explained by geographic distance, we performed a Mantel test (Mantel, 1967) using Euclidean geographic distance and assemblage similarity among sites. All analyses were done in R environment using the packages “vegan” (Oksanen et al., 2016), “ca” (Nenadic & Greenacre, 2007) and “MASS” (Venables & Ripley, 2002).

### 2.7 Functional diversity

To examine changes in bat functional diversity among treatments and habitats, we classified the species captured into guilds (Kalko, 1997; Schnitzler & Kalko, 1998) based on their foraging modes (i.e., aerial or gleaning), diet (i.e., carnivores, frugivores, insectivores, nectarivores, omnivores, piscivores, or sanguivores), and preferred habitat (i.e., background cluttered space, highly cluttered space, uncluttered space; Supporting Information Table A1). The term clutter “represents mechanical as well as perceptual problems for bats” (Kalko, 1997). In addition to this guild classification, we differentiated the species in the “highly cluttered space‐gleaning‐frugivore” guild into shrub (understory) or canopy species based on their use of forest strata following other studies (Aguirre, Montano‐Centellas, Gavilanez, & Stevens, 2016; Sampaio, Kalko, Bernard, Rodriguez‐Herrera, & Handley, 2003; Supporting Information Table A1). We characterized the functional diversity using observed and estimated guild richness with the nonparametric estimator Chao 1. In addition, following Aguirre et al. (2016) we estimated the inverse of Simpson's index based on the number of species within each guild. To analyze relative importance of guilds within each level (i.e., treatments, habitats within treatments, and RS sites), we generated rank richness distribution curves, based on the proportion of species per guild, and compared the proportional abundance of guilds.

## 3 RESULTS

In a total of 3,456 mist‐net hours across 48 nights, we captured 43 species and 1,237 individuals from the family Phyllostomidae (Supporting information Table A2). Four species—*Phyllostomus hastatus* (199 captures), *Artibeus planirostris* (171), *Carollia perspicillata* (155), and *Carollia brevicauda* (121)—accounted for 52.2% of the total captures.

### 3.1 Effectiveness of the sampling

**Figure 2:**
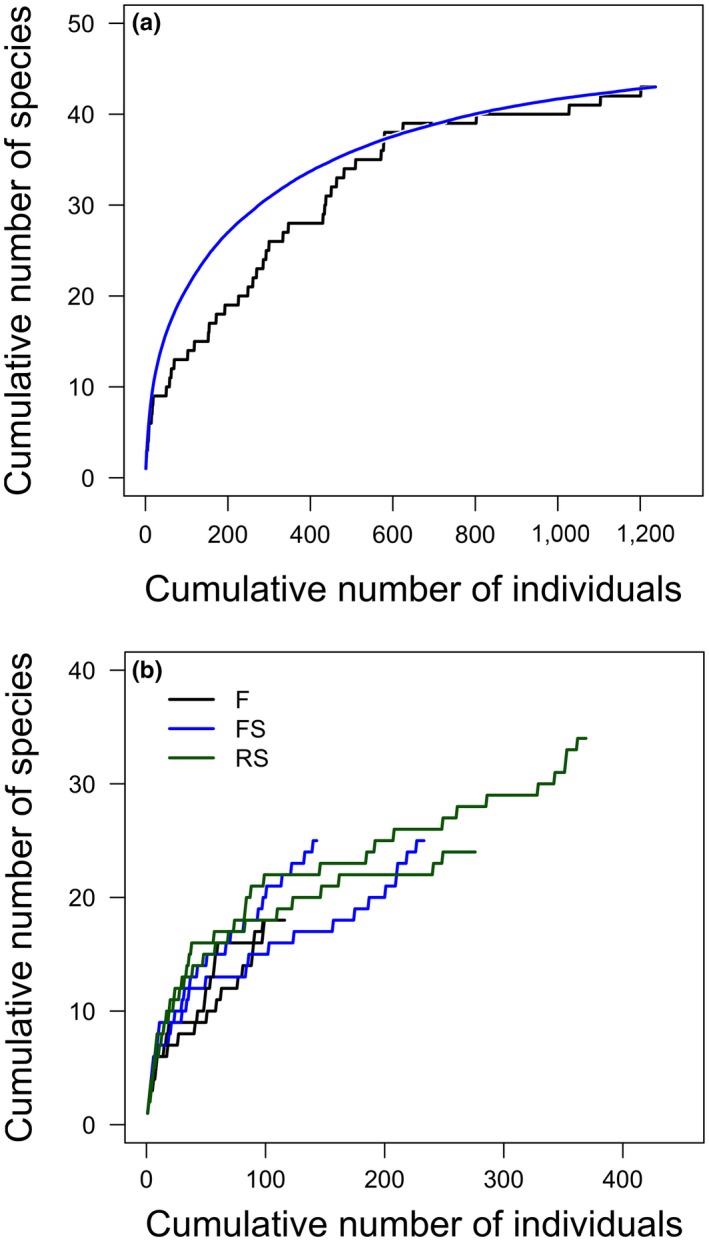
Species accumulation curves. (a) All sites combined, and a smoothed curve; and (b) Individual sites: F: forest; FS: stream habitat in forest; RS: riparian forest strips in open areas cleared of forest

When including all captures, the cumulative number of Phyllostomidae species appeared to reach an asymptote, indicating that few bat species would likely be added with additional sampling effort (Figure 2a). When we observed the accumulation curves for each site, only RS sites appeared to be reaching an asymptote in number of species recorded; thus, additional sampling would likely add more species to all sites, especially the FS and F sites (Figure 2b).

### 3.2 Species richness

Overall, we captured 23 species of phyllostomids in F sites, 33 in FS sites, and 36 in RS sites. Estimated number of bat species across all sites was 47.5 ± 4.80 (Chao 1) and 49.0 ± 2.45 (Jackknife 1) (Table 1). The estimated range of species richness in each site varied from 28.5–82.9 (Chao 1) and 24.9–48.0 (Jackknife 1) (Table 1). Among treatments, RS had the highest observed and estimated number of species, while RS1, the site that was recently cleared for palms, had the greatest observed and estimated number of species and captures followed by FS1. In contrast, RS2 showed the lowest observed and estimated species richness among sites. Observed and estimated numbers of species for different habitats per site are shown in Supporting Information Table A3.

**Table 1:** Observed and estimated number of species captured by site

Treatment Site	Observed species	Chao 1	Chao 1−*SE*	Jackknife 1	Jackknife 1−*SE*	Individuals captured
F	23	33.62	10.22	30.96	2.81	216
FS	33	42.97	8.34	42.97	3.15	376
RS	36	46.11	9	44.99	2.99	645
F1	18	30.13	13.03	24.93	2.62	100
F2	18	30.15	13.04	24.94	2.62	116
FS1	25	74.78	59.33	34.96	3.15	233
FS2	25	40.89	16.38	32.94	2.81	143
RS1	34	82.87	43.88	47.96	3.73	369
RS2	24	28.48	4.79	29.98	2.44	276
Total	43	47.5	4.8	49	2.45	1,237

Note

*SE*, standard error.

**Figure 3:**
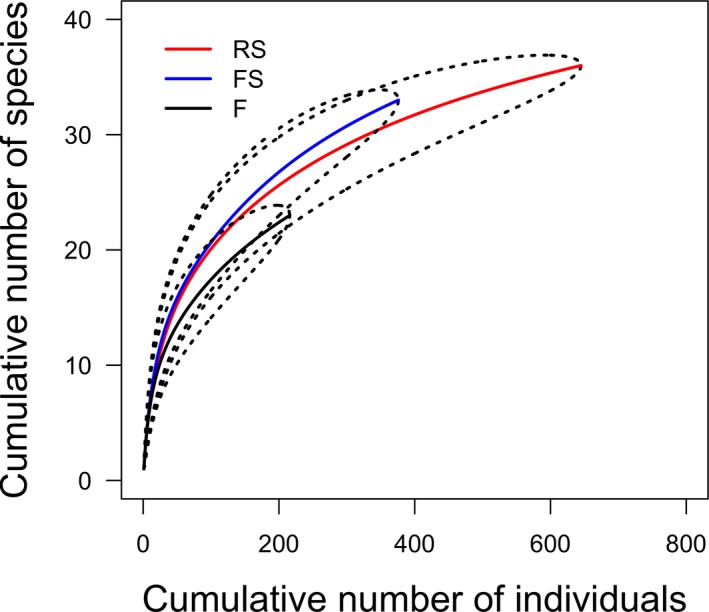
Individual‐based rarefaction curves for the bat species registered within the three treatments. Dotted and dashed lines represent 95% confidence intervals. F: forest; FS: stream in forest; RS: riparian forest strips in open areas cleared of forest

**Figure 4:**
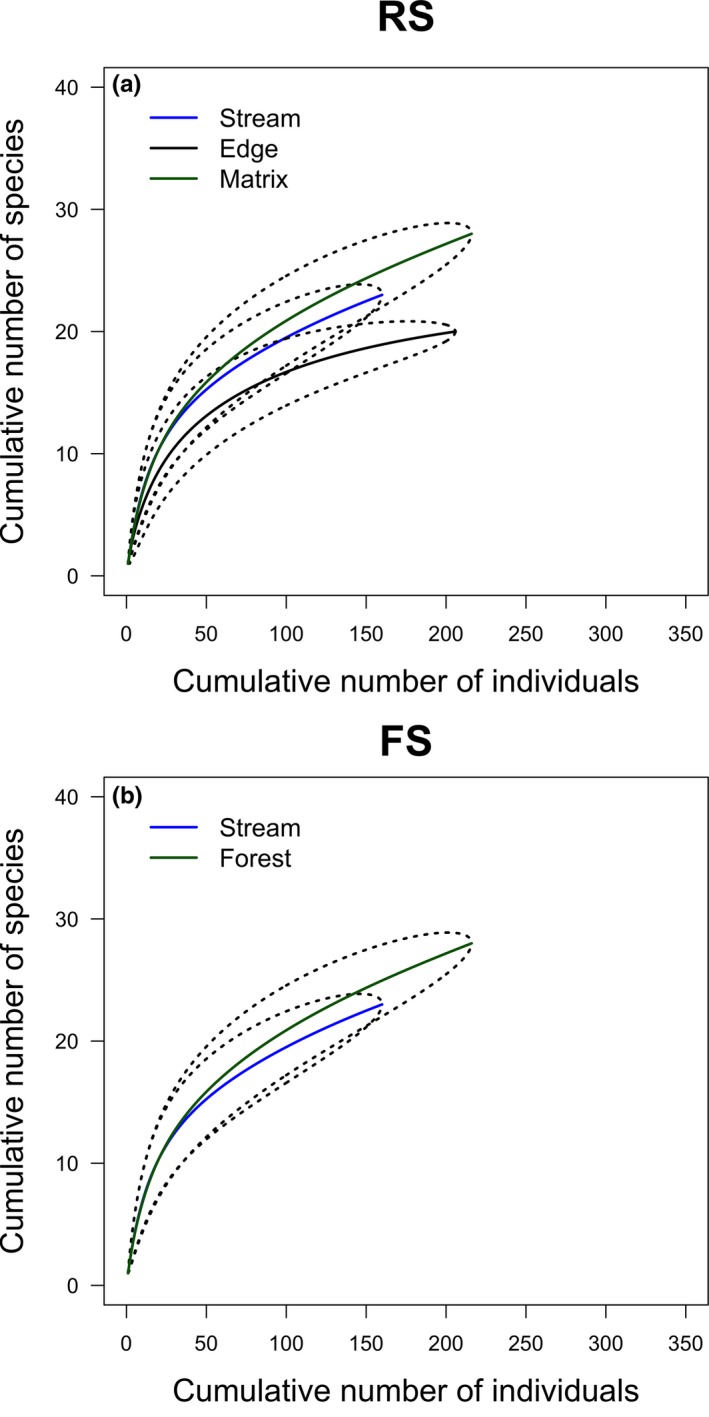
Individual‐based rarefaction curves for the bat species in different habitats. (a) RS: riparian forest strips in open areas cleared of forest, (b) FS: stream in forest. Dotted and dashed lines represent 95% confidence intervals

Although the estimated number of species showed differences at the levels of analysis, rarefaction results were not significant. Using the lowest capture total among treatments (216 individuals in treatment F), we found that species richness did not differ among treatments using rarefaction (Figure 3); this result also held at the site level (Supporting Information Figure A1). The number of species also tended not to differ among habitats (Figure 4a, b) when using rarefaction techniques that control for the lowest number of bats captured in a habitat, but matrix habitats did have more species captured than edge habitats in RS sites (Figure 4a).

We examined how bat captures differed among habitats in RS1 and RS2 (Supporting Information Figure A2). All three species of the genus *Carollia* were found in all three habitats at both sites. *Phyllostomus hastatus* and *Desmodus rotundus* were captured mainly in stream and edge habitats, and only rarely in the matrix. *Lophostoma silvicolum* was captured in all three habitats in RS1 but only once in the stream habitat in RS2. *Rhynophylla pumilio* was absent in the cleared matrix in RS2, but was captured in the cleared matrix in RS1.

### 3.3 Species abundance distribution

**Figure 5:**
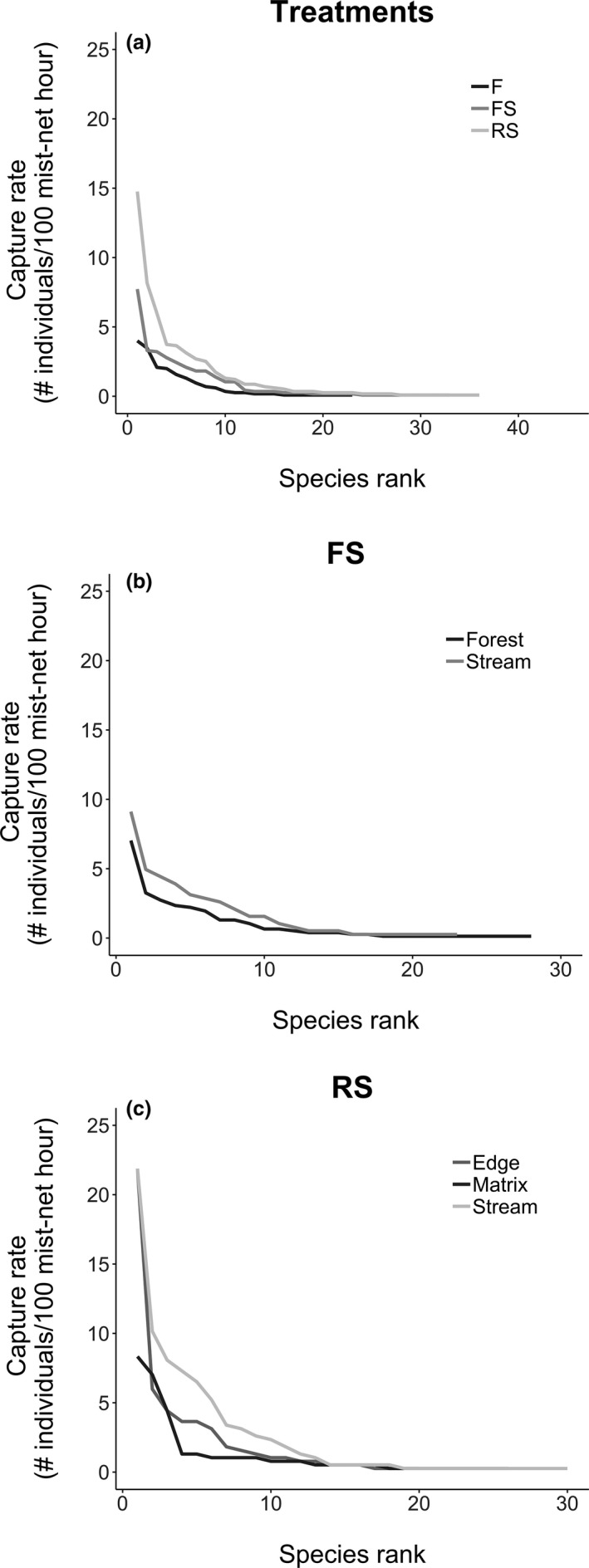
Rank‐abundance distributions. (a) By treatments and by habitat in: (b) FS: stream in forest and (c) RS: riparian forest strips in open areas cleared of forest

**Figure 6:**
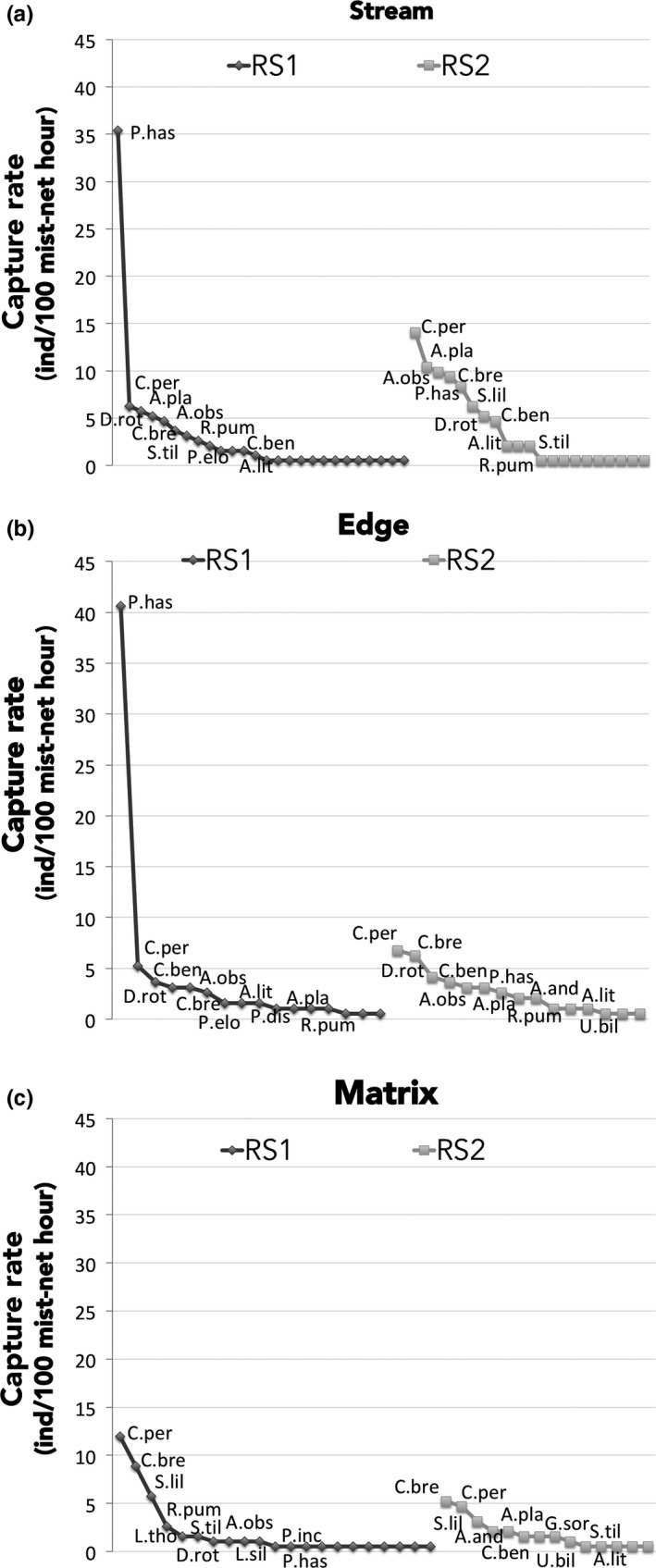
Species rank‐abundance curve at each habitat type in the RS sites. (a) stream, (b) edge, (c) matrix. *P. has*: *Phyllostomus hastatus*; *C. per*: *Carollia perspicillata*; *A. pla*: *Artibeus planirostris*; *A. obs*: *Artibeus obscurus*; *C. bre*: *Carollia brevicauda*; *D. rot*: *Desmodus rotundus*; *R. pum*: *Rhinophylla pumilio*; *S. til*: *Sturnira* tildae; *S. lil*: *Sturnira lilium*; *C. ben*: *Carollia benkeithi*; *A. lit*: *Artibeus lituratus*; *P. elo*: *Phyllostomus elongatus*; *U. bil*: *Uroderma bilobatum*; *L. tho*: *Lonchophylla thomasi*; *G. sor*: *Glossophaga soricina*; *A. and*: *Dermanura anderseni*; *P. inc*: *Platyrrhinus incarum*; *L. sil*: *Lophostoma silvicolum*. RS = riparian forest strips in open areas cleared of forest

Overall, the shape of the rank‐abundance curves did not differ among the three treatments (sites combined, Figure 5a), six sites (all habitats combined, Supporting Information Figure A3), nor among habitats within RS (all Kolmogorov–Smirnov two‐sample tests, *p* > 0.05). Only in FS sites did the shape of rank‐abundances curves differ between forest and stream habitats (*D* = 0.39, *p* = 0.047) (Figure 5b, c). Despite the absence of significant differences in the shape of the rank‐abundance curves, some patterns stand out. For example, which species were most abundant and the number of rarely captured species which define the length of the tail, differed among treatments. Further, when comparing the habitats within RS treatment (Figure 6a–c), the rank‐abundance curve for the stream habitat at RS1 had a longer tail indicating more species captured only a few times when compared to RS2 (Figure 6a). In RS1 and RS2 sites, the omnivore *Phyllostomus hastatus* is largely driving the differences observed in stream and edge habitats (Figure 6a, b). With the exception of *P. hastatus* at RS1, the assemblage in both RS sites was dominated by *Carollia perspicillata*, *C. brevicauda*, *Artibeus planirostris,* and *A. obscurus*.

### 3.4 Assemblage composition

**Figure 7:**
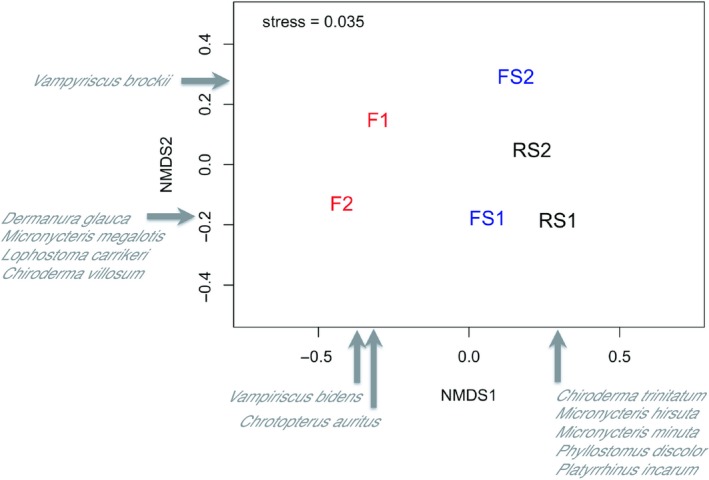
Non‐multidimensional scaling analysis (NMDS) in 2‐dimensional space at the site level based on the phyllostomid bat assemblage composition using relate abundance data from six sites at Caynarachi, San Martin—Peru. *F*: forest; FS: stream in forest; RS: riparian forest strips in open areas cleared of forest

As species’ capture rates varied greatly, we standardized the data using Wisconsin double standardization (Bray & Curtis, 1957). We found that bat assemblages from F sites were distinct from treatments FS and RS, likely because of the presence of stream habitats in the latter two treatments (Figure 7; see also Supporting Information Table A4), although there was no difference among treatments (PerMANOVA *F* = 1.33, *p* = 0.2). Species characteristic of sites with stream habitat (i.e., species with high positive loadings on axis 1) included *Chiroderma trinitatum*, *Micronycteris hirsuta*, *M. minuta*, *Phyllostomus discolor*, *Platyrrhinus incarum* (Figure 7, Supporting Information Table A4).

**Figure 8:**
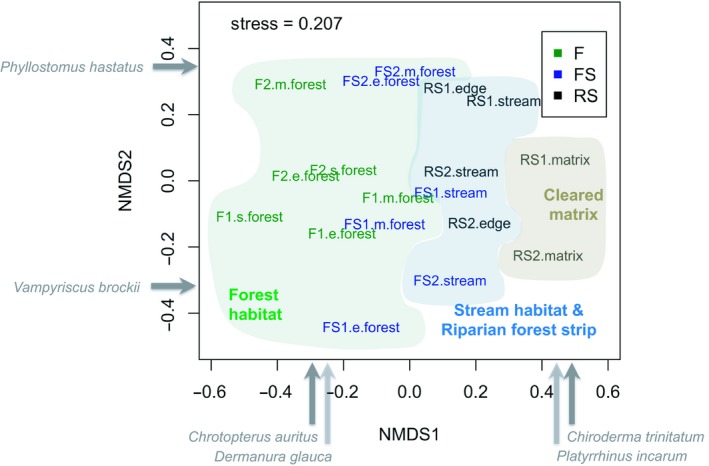
Non‐multidimensional scaling analysis (NMDS) in 2‐dimensional space at the fine scale based on the phyllostomid bat assemblage and abundance at Caynarachi, San Martin—Peru: eighteen transects. s.forest = transects in “stream” position in F sites, e.forest = transects of forest habitat in “edge” position in F and FS sites, m.forest = transects of forest habitat in “matrix” position in forest (F) and stream habitat in forest (FS) sites. RS = riparian forest strips in open areas cleared of forest. Polygons show how the transects group themselves in forest habitat, stream and riparian forest strip habitat, and cleared matrix and are drawn to connect similar units

Considering the assemblage similarity among habitats at different sites, we found that the first axis separated the habitats with riparian zones (i.e., stream in forested sites, stream and edge in riparian forest strips in cleared sites) generally being intermediate between forest and cleared matrix (PerMANOVA *F* = 2.2 and *p* < 0.05; Figure 8, Supporting Information Table A5).

At the treatment level, all species registered at F were also found in FS and/or RS, with the exception of *Chrotopterus auritus*. FS sites contained three unique species (*Vampyriscus brockii*, *Dermanura glauca*, *Micronycteris megalotis*), and all were captured only within forest habitat. RS sites contained seven unique species (*Dermanura *cf*. cinerea*, *Chiroderma trinitatum*, *Lionycteris spurelli*, *Micronycteris hirsuta*, *M. minuta*, *Platyrrhinus incarum*, *Phyllostomus discolor*) with none of them restricted to the cleared matrix. In contrast, seven species were missing at RS sites, including *Mimon crenulatum* (recently revised to *Gardenycteris crenulatum*, Hurtado & Pacheco, 2014), *Vampyriscus bidens, *and *Vampyressa thyone* in addition to the unique species from F and FS treatment. Considering those species captured more than once, only two species were restricted to a particular habitat; *Lophostoma carrikeri* and *Sturnira* cf. *luisi* were captured only alongside streams in FS and RS (Supporting Information Figure A2).

When RS sites are compared, we captured 12 species in RS1 that were absent at RS2; capture rate also was higher in RS1 than RS2. Most of these 12 species were captured only alongside streams although *Chiroderma trinitatum* and *C. villosum* were only captured in the RS1 cleared matrix. Only two species captured in RS2 were absent in RS1 (*Dermanura *cf*. cinerea*, *Lionycteris spurelli*). We found no evidence that geographic distance between sites explained similarity in bat species composition (Mantel test, correlation = 0.14, *p* = 0.279).

### 3.5 Functional diversity

Phyllostomid bat species captured correspond to seven guilds: highly cluttered space‐gleaning‐carnivores (Car), highly cluttered space‐gleaning‐canopy‐frugivores (CanFru), highly cluttered space‐gleaning‐shrub‐frugivores (ShrFru), highly cluttered space‐gleaning‐insectivores (HCIns), highly cluttered space‐gleaning‐nectarivores (Nec), highly cluttered space‐gleaning‐omnivores (Omn), and highly cluttered space‐gleaning‐sanguivores (San). Hereafter for simplicity, we will refer to the guilds by their diet and, when appropriate, strata (i.e., carnivores, canopy frugivores, insectivores, nectarivores, omnivores, sanguivores, and shrub frugivores) since all guilds registered were highly cluttered space‐gleaning bats.

We did not observe differences in functional diversity at the treatment level. However, differences were evident at the habitat level depending on the treatment. The observed functional richness varied from five to seven guilds per habitat within FS and RS treatments, and from four to seven guilds among habitats within RS sites (Table 2). Bats functional diversity was higher in F than in other treatments; stream habitat in the RS treatment had higher functional diversity than those in FS and RS treatments, while matrix habitat in RS1 was higher than matrix in RS2 (Table 2).

**Table 2:** Functional diversity of the bat assemblages based on the observed diversity of guilds (Observed Functional Richness and Inverse of Simpson Index based on the number of species within each guild) and estimated functional diversity (Chao 1) by treatment, by habitat, and by age of clearing

Level	Observed Functional Richness	Chao 1	Chao 1−*SE*	Inverse of Simpson Index	Number of captures
Treatment					
F	7	7.50	1.32	4.60	216
FS	7	7.00	0.46	3.72	376
RS	7	7.00	0.46	4.23	645
Habitats					
FS stream	5	5.00	0.00	3.09	160
FS forest	7	7.00	0.46	3.63	216
RS stream	7	7.50	1.32	4.13	309
RS edge	6	6.00	0.00	4.08	206
RS matrix	6	6.00	0.00	3.75	130
Clearing Ages					
RS1 stream	7	7.99	2.22	4.17	156
RS1 edge	6	6.00	0.45	4.27	132
RS1 matrix	6	6.00	0.00	4.28	81
RS2 stream	6	6.50	1.31	3.47	153
RS2 edge	5	5.00	0.44	3.17	74
RS2 matrix	4	4.00	0.42	2.58	49

**Figure 9:**
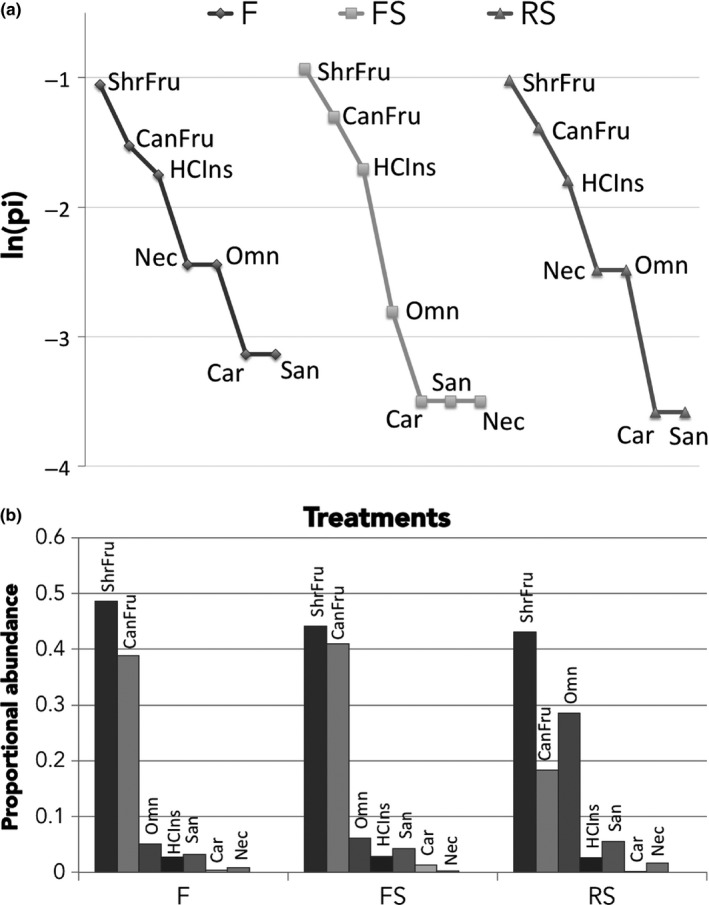
(a) Rank richness distribution curves of guilds by treatment, (b) proportional abundance of each guild by treatment. (HCIns) highly cluttered space‐gleaning‐insectivores, (Car) highly cluttered space‐gleaning‐carnivores, (San) highly cluttered space‐gleaning‐sanguivores, (CanFru) highly cluttered space‐gleaning‐canopy‐frugivores, (ShrFru) highly cluttered space‐gleaning‐shrub‐frugivores, and (Nec) highly cluttered space‐gleaning‐nectarivores, (Omn) highly cluttered space‐gleaning‐omnivores. ln = natural logarithm, pi = proportion of the total number of species registered at the treatment that correspond to a specific guild at that treatment

Across all treatments, the guilds that occupied the first rank positions based on their species richness were shrub frugivores followed by canopy frugivores and insectivores (Figure 9a). In terms of proportional abundance, F and FS treatments showed similar patterns where shrub frugivores and canopy frugivores again dominated the assemblage but they were followed by omnivores instead of insectivores. RS treatment, however, had fewer canopy frugivores and more omnivores (Figure 9b).

**Figure 10:**
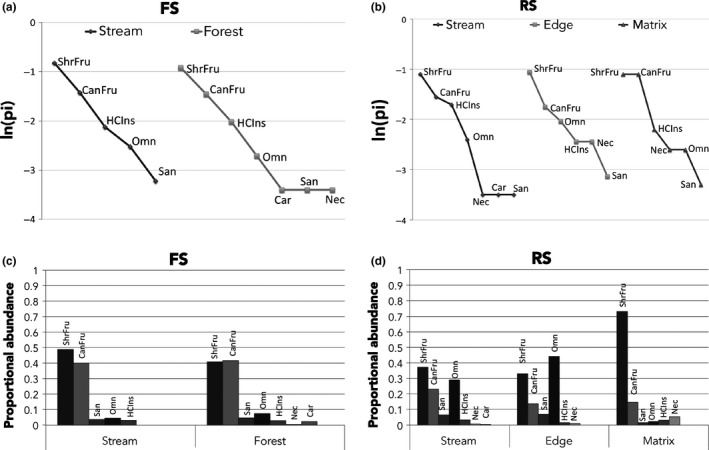
Rank richness distribution curves of guilds by habitat. (a) stream habitat in forest (FS), (b) riparian forest strips in open areas cleared of forest (RS), (c) proportional abundance of the guilds in FS habitats, (d) proportional abundance of the guilds in RS habitats. (HCIns) highly cluttered space‐gleaning‐insectivores, (Car) highly cluttered space‐gleaning‐carnivores, (San) highly cluttered space‐gleaning‐sanguivores, (CanFru) highly cluttered space‐gleaning‐canopy‐frugivores, (ShrFru) highly cluttered space‐gleaning‐shrub‐frugivores, (Nec) highly cluttered space‐gleaning‐nectarivores, (Omn) highly cluttered space‐gleaning‐omnivores. ln = natural logarithm, pi = proportion of the total number of species registered at each habitat by treatment that correspond to a specific guild at that treatment

Both habitats in FS treatment had similar rank richness curves, but the forest habitat curve contained additional guilds with few species (Table 2, Figure 10a). Notwithstanding the observed patterns in rank richness distribution, both frugivore guilds dominated FS habitats in terms of their proportional abundance, while all other guilds were represented by relatively few individuals (Figure 10c). In RS, all habitats had similar rank richness distribution curves (Figure 10b); but the patterns of proportional abundance differed among them. In stream and edge habitats, shrub and canopy frugivores, as well as omnivores, dominated the assemblages (Figure 10d); in both cases, these three guilds account for 89.3% and 90.8% of the total abundance in each habitat, respectively. However, in matrix habitat, shrub frugivores alone dominated with 73% of the total abundance (Figure 10d).

**Figure 11:**
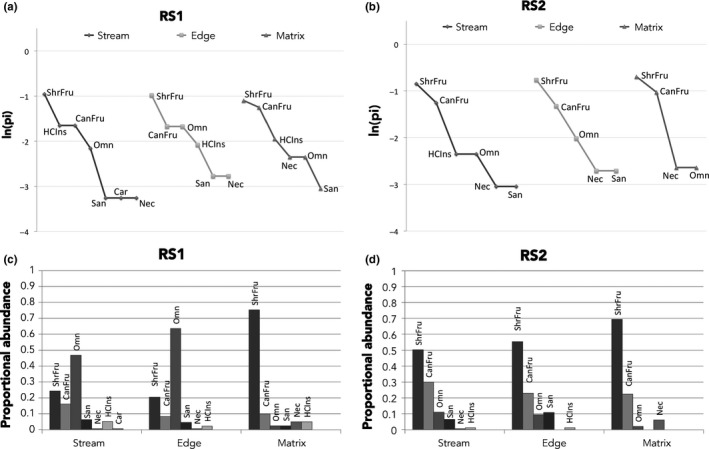
Rank richness distribution curves of guilds at (a) RS1 and (b) RS2. Proportional abundance of the guilds in (c) RS1 habitats and (d) RS2 habitats. (HCIns) highly cluttered space‐gleaning‐insectivores, (Car) highly cluttered space‐gleaning‐carnivores, (San) highly cluttered space‐gleaning‐sanguivores, (CanFru) highly cluttered space‐gleaning‐canopy‐frugivores, (ShrFru) highly cluttered space‐gleaning‐shrub‐frugivores, (Nec) highly cluttered space‐gleaning‐nectarivores, (Omn) highly cluttered space‐gleaning‐omnivores. ln = natural logarithm, pi = proportion of the total number of species registered at the treatment that correspond to a specific guild at that treatment

When comparing RS1 and RS2 sites, stream habitats had the highest number of guilds at both sites, with seven and six guilds, respectively (Table 2, Figure 11). RS1 had similar rank species distribution curves for the three different habitats (Figure 11a), but the stream habitat in RS2 had one and two more guilds than edge and matrix habitats, respectively (Figure 11b). Moreover, matrix habitat in RS2 showed the lowest number of guilds (four), with shrub and canopy frugivores dominating the assembly (Figure 11b). In terms of proportional abundance, RS1 had a dominance of omnivores in stream and edge habitats, while matrix habitat was dominated by shrub frugivores (Figure 11c). At RS2, shrub frugivores dominated the assemblages in all habitats, especially matrix where the other guilds were less abundant (Figure 11d).

## 4 DISCUSSION

Our results support the hypothesis that riparian forest strips are important conservation assets in agricultural landscapes, during early stages of the clearing process. High capture rates and high numbers of bat species, including species rarely captured, found in the riparian forest strips support our assertion. Indeed, riparian forest strips surrounded by recently cleared areas maintained 75% of the bat species registered in forest and stream habitats in F and FS sites, and, in addition, contained six species not found in these forested habitats. Moreover, functional diversity of bat species in riparian forest strips closely matched that found in forested areas, suggesting that maintenance of ecological services in the landscape is favored by retention of riparian strips. Despite the conservation value of riparian forest strips (RS), however, seven species found in forest sites (F and FS) were missing in these RS assemblages. In the following paragraphs, we discuss why riparian strips are effective at maintaining bat species and elaborate further on their conservation value.

Habitat appears to be an important factor that contributes to explaining distribution of bats in San Martín (Figure 8). Further, diversity of bats among treatments likely reflects habitat heterogeneity in these sites (i.e., presence of stream and forest habitats vs. forest habitats only; Figures 3 and 4). In previous studies, forest habitats also were shown to have lower bat species richness and abundance when compared with edge and nonforest habitats (de la Pena‐Cuellar et al., 2015; Martins, Willig, Presley, & Marinho‐Filho, 2017). Habitat differences are thought to promote beta diversity, as changes in vegetation structure result in turnover of bat species (Laurance et al., 2002). Further, fine‐scale vegetation factors in riparian forest influence activity of insectivorous bats, affecting their flight and foraging abilities (Ober & Hayes, 2008).

In accordance to our predictions, we found that the bat assemblage in riparian forest strips was more similar to the assemblage in stream habitat, suggesting riparian strips are providing habitat to bat species. Other studies have highlighted the importance of riparian forest habitats due to their high concentration of food resources (insects and plants), and their role in providing water, and potential roost sites (Galindo‐Gonzalez & Sosa, 2003; Grindal, Morissette, & Brigham, 1999). Riparian vegetation was also considered as a refuge for bat species (de la Pena‐Cuellar et al., 2015) and other taxa (Darveau et al., 2001), facilitating plant regeneration processes and plant population maintenance (Galindo‐Gonzalez & Sosa, 2003).

Also, our results show RS sites had higher capture rates than other treatments, a good representation of the species at FS and F habitats, and even species absent from the latter, which may be an indication of riparian forest strips serving as movement corridors, other of our predictions. Indeed, most individuals were captured along streams in RS sites, thus supporting the conservation value of riparian forest strips as corridors. Previous studies found riparian vegetation is crucial to ensure bat mobility across human‐modified landscapes (Galindo‐Gonzalez & Sosa, 2003). Other studies also observed higher richness of phyllostomid species in riparian forest when compared to open areas or continuous mature forest (Arriaga‐Flores et al., 2012; de la Pena‐Cuellar et al., 2015; Lourenco et al., 2014). This may also indicate that riparian forest strips add value when compared to forest corridors without streams. In essence, riparian forest strips appear to be more “species‐rich” than forest corridors without streams. Increased species richness in these forest strips may result from increased capture probability due to narrow forested area, or may represent an “ark” effect as species and individuals accumulate in forest areas following recent forest clearing. Support for the latter may partially explain why the riparian forest strip in an older cleared area had lower species richness, assuming some “relaxation” of individuals and species had occurred. Further, the small area of the riparian forest strip may facilitate bat captures, however, one of the riparian forest strips sites was fourth in number of species, serving as evidence to deny this possibility.

High species richness in RS sites was also accompanied by considerable variation in species’ capture rates with few very abundant species, several common species and many captured only a few times. This pattern is typical for phyllostomid bat species in Neotropical areas (Arriaga‐Flores et al., 2012; Estrada, Coates‐Estrada, Meritt, Montiel, & Curiel, 1993). The number of species captured rarely may reflect their use of riparian strips as movement corridors among larger forest patches, rather than permanent occupancy in riparian strips.

Bat assemblages in forest habitat and the cleared matrix were a subset of bats found in riparian forest strip habitat (stream & edge habitat at RS treatments). Bat species with low captures in the former habitats but abundant in riparian strips included medium‐bodied species of the genus *Artibeus* (*A. planirostris*, *A. obscurus*). Similarly, *Carollia benkeithi*, *Phyllostomus hastatus*, *P. elongatus,* and *Desmodus rotundus* had lower capture rates in the cleared matrix than in riparian forest strips at both RS sites. The low captures of *Artibeus planirostris* and *Phyllostomus hastatus* in the cleared matrix habitat at the RS sites, however, might be due to methodological constraints. Both species, which are considered to be canopy foragers (Cisneros, Fagan, & Willig, 2015; Klingbeil & Willig, 2009; Ramos‐Pereira, Marques, & Palmeirim, 2010; Rex, Michener, Kunz, & Voigt, 2011), may commute among sites via flying above level of mist‐nets and were not captured in the matrix.

Despite the apparent conservation value of riparian forest strips to a number of bat species, the presence of bat species in cleared matrix habitat suggests that some species do cross nonforested matrix, at least at the scale studied here. For example, *C. brevicauda* and *C. perspicillata* were abundant across sites and habitats, and were frequently captured in the matrix. *C. perspicillata *reportedly flies long distances while foraging (Heithaus & Fleming, 1978), which may allow it to use different elements in the landscape more readily than other species. *Sturnira lilium*, *Lonchophylla thomasi*, and *Uroderma bilobatum* also were frequently captured in matrix habitat. In previous studies, *U. bilobatum *and *S. lilium* were found to be associated with riparian forest (de la Pena‐Cuellar et al., 2015); *S. lilium* also were found readily in modified forested landscapes, flying across open areas to move among forest patches (Cisneros et al., 2015; Loayza & Loiselle, 2008).

Regarding functional diversity, even though the number of guilds was similar across treatments, there were differences in functional diversity at the habitat level, which provide insights regarding the conservation value of riparian forest strips. Relative to matrix habitats, stream and edge habitats in RS sites were functionally more diverse, and thus, more likely to maintain ecological functions or services in the landscape; this supports our hypothesis about the potential conservation value of riparian forest strips.

Riparian forest strips, however, are not likely to serve as refuges or corridors for all species. For example, *Chrotopterus auritus*, a large carnivorous bat, was only registered in forest sites (F). *C. auritus* is sensitive to perturbation and is generally found in undisturbed forest (Castro‐Arellano, Presley, Saldanha, Willig, & Wunderle, 2007; Fenton et al., 1992; Gorresen & Willig, 2004). Similarly, *Chiroderma trinitatum* and *C. villosum* were only found in the matrix habitat at RS sites. Species of *Chiroderma *forage mainly in forest canopies (Kalko & Handley, 2001; Rex et al., 2011). *Chiroderma villosum* is found mainly in modified landscapes (Cisneros et al., 2015) and is highly mobile (Meyer & Kalko, 2008). Each of these three species is considered rare, and whether their absence reflects a sampling bias or that riparian forest habitats are unsuitable habitat remains unclear.

Although our study was not designed to examine the value of riparian forest strips over time, our results suggest that time since clearing may be related to how well riparian forest strips conserve biodiversity. The observed decreases in captures and diversity with time since creation was explained as a crowding effect for Amazonian forest fragments (Bierregaard, Lovejoy, Kapos, Dossantos, & Hutchings, 1992; Debinski & Holt, 2000). In this study, RS2 site had only a subset of phyllostomid bats shortly after the surrounding forests were cleared likely indicating the occurrence of crowding effects that dissipated with time. These results, however, may also reflect local differences between the two sites. For future studies, a time series analysis is highly recommended to evaluate the effects of crowding. Riparian forest strips may also be subject to edge effects and degrade over time (Pereira et al., 2018). Examination of how edge effects may affect bat diversity and abundance in riparian strips is needed. For other taxon groups, the disturbance of riparian forests showed overall negative effects on diversity (e.g., benthic assemblages, Iwata, Nakano, & Inoue, 2003; birds and mammals, Lees & Peres, 2008). In addition, future studies that include a comparison with open agricultural land with and without the presence of streams would be valuable. However, there is strong evidence that in the absence of forest habitat, stream areas without riparian forest strips had lower diversity (birds, Triquet, McPeek, & McComb, 1990; fish, Giam et al., 2015, Jones, Helfman, Harper, & Bolstad, 1999, Lobon‐Cervia, Mazzoni, & Rezende, 2016). Moreover, evaluating ecological parallels with natural savanna‐forest systems may help to enrich the discussion of the conservation value of riparian forest strips in agricultural landscapes. Tropical savannas are biodiversity hotspots (Myers, Mittermeier, Mittermeier, Fonseca, & Kent, 2000) known for their high bat diversity (Bernard & Fenton, 2002; Lim & Lee, 2018; Morales‐Martinez, Rodriguez‐Posada, Fernandez‐Rodriguez, Calderon‐Capote, & Gutierrez‐Sanabria, 2018). Forest formations (i.e., riparian forests or gallery forests, forest islands) within savannas are considered as key elements to maintain bat diversity in the landscape (Aguirre, 2002; Lima, Varzinczak, & Passos, 2017; Morales‐Martinez et al., 2018). Comparing savanna ecosystems with study systems like the one analyzed here provide insights regarding underlying ecological processes important to maintain diversity.

In this study, the width of the riparian forest strip was constant (~25 m on either side of the stream). Likely the degree to which riparian forest strips effectively mitigate forest disturbance will be dependent on their width. A comparison of the effects of different widths of riparian strip habitat on maintenance of biodiversity is needed to guide decision‐makers and managers. In previous studies examining widths of riparian forest strips, Davies and Nelson (1994) found width to strongly influence stream production. Riparian forest strip widths <10 m showed changes in algae, macroinvertebrates and fish in streams, while widths >30 m were found to be effective in buffering streams from such changes in the short term (macroinvertebrates, fish and algae, Davies & Nelson, 1994; macroinvertebrates, Newbold et al., 2015). In addition, widths of riparian habitats have been shown to affect terrestrial species (birds, Hagar, 1999, Mitchell et al., 2018; dung beetles, Gray et al., 2014; but see Darveau et al., 2001, small mammals). A study in the Brazilian Amazon showed that most of the variation in bat composition occurs up to 114 m from the streams (Pereira et al., 2018), thus riparian forest strips of ~25 m wide would conserve just a fraction of the bat assemblage. In this study, 25 m wide forest strips appeared to provide conservation value, but such value would likely improve by increasing the width of forest remaining alongside streams. Management decisions about width of forest strips should consider the likely impacts of edge effects, as these edge effects are likely to cause changes in the abundance of most forest species present in the strips (Ries, Fletcher, Battin, & Sisk, 2004). For example, edge effects can result in significant changes to vegetation structure as trees along edges exposed to strong winds suffer greater mortality (Saunders et al., 1991). Riparian forest strips that are wider may offer some resilience to edge effects and better maintain conservation value. Determining what are optimum widths to balance needs of production and conservation are questions that need to be assessed as a function of the surrounding land use (e.g., widths needed in open pasture habitats may differ from those in palm plantations).

An important consideration for our study is that it was conducted in a landscape with agricultural activity and forested areas. Riparian forest strips may not be effective mitigation measures if they are not connected to areas of continuous or well‐maintained forest as riparian forest strips alone will not contain adequate resources to support most bat species.

Finally, our study examined only the very early stages following land clearing. As palm plants grow and provide increased vegetative structure, and presumably provide additional resources and cover, the assemblage of phyllostomid bats using the matrix may change. Such changes in bat abundance and composition have been shown with regrowth of secondary forest around forest fragments (Gascon et al., 1999; Rocha et al., 2018). How changes in the matrix affect the conservation value of riparian forest strips is another area ripe for future study.

## 5 CONCLUSIONS

This study highlights the conservation value of riparian forest strips in agricultural landscapes. These strips likely serve as both habitat and movement corridors for some bat species. Although some rare and perturbation‐sensitive species appear to be absent, the strips harbored a large proportion of the original bat assemblage. Where the government encourages private investment for agriculture and forest clearing, more emphasis should be given to adopting best practices and conservation planning to maintain forest diversity as established in Peruvian environmental policy (Decreto Supremo Nº 012‐2009‐MINAM, www.minam.gob.pe/wp-content/uploads/2013/09/ds_012-2009-minam.pdf). Even though riparian strips in this study may be considered narrow (i.e., 25 m width), they showed an important conservation value during the first stages after forest clearing. Riparian forest strips that occurred in areas surrounded by recently cleared lands maintained 75% of the bat species registered in forested habitats. In addition, they maintained species from all guilds registered in the study. The results of this study provided basic data to evaluate the efficacy of maintaining these landscapes features for mitigating impacts of agricultural development on biodiversity. This study also provides information for defining widths of riparian forest strips that government authorities will require to land owners and private companies. Promoting the enforcement of laws that require the maintenance of riparian forest strips is essential for conserving biodiversity and maintaining functional ecosystems. However, we caution relying solely on the maintenance of riparian forest strips, since its effectiveness will depend on the landscape context and the presence of nearby forested areas.

## CONFLICT OF INTEREST

Authors have no conflict of interest to declare.

## AUTHOR CONTRIBUTIONS

F. Carrasco‐Rueda collected data from the field, conducted analyses, and was the lead on writing the manuscript. Both authors contributed in the conception and design of the study and contributed to the writing of the manuscript.

## Data Availability

Bats capture data that supports the results of this article has been deposited to a public repository: Dryad https://doi.org/10.5061/dryad.3td78sk.
