# Supplementary material for: Do riparian forest strips in modified forest landscapes aid in conserving bat diversity?
Source: Ecol Evol. 2019 Mar 27;9(7):4192–209. doi: 10.1002/ece3.5048 (PMC6467860; doi:10.1002/ece3.5048)
Supplement: Supplementary file 1 [file ECE3-9-4192-s001.docx]

**Supplementary Information**

**RESUMEN**

Las prácticas agrícolas conllevan a la pérdida de recursos naturales y biodiversidad. Mantener bosques a lo largo de arroyos (franjas de bosque ripario) ha sido utilizado como un mecanismo para minimizar el impacto de la apertura de bosque para agricultura en la biodiversidad. Para probar la contribución de las franjas de bosque ripario a la conservación de la biodiversidad en paisajes productivos, seleccionamos a los murciélagos como un sistema modelo de biodiversidad y examinamos dos dimensiones: diversidad taxonómica y diversidad funcional. Comparamos la diversidad y composición de murciélagos en bosques y en franjas de bosque ripario angostas rodeadas por áreas abiertas para agricultura. Los sitios de bosque incluyeron replicas con y sin hábitat de arroyo. Probamos la hipótesis que las franjas de bosque ripario proveen un potencial valor de conservación brindando hábitat y sirviendo como corredores de movimiento para especies de murciélagos de bosque. Las franjas de bosque ripario mantuvieron 75% de las especies de murciélagos registradas en los hábitats de bosque. Encontramos que los ensamblajes en los sitios con franjas de bosque ripario estaban dominados por pocas especies muy abundantes e incluyeron varias especies con poca abundancia. Los ensamblajes de especies de murciélagos fueron más similares entre sitios con arroyos que entre los sitios de bosque si hábitat de arroyo. Estos resultados resaltan la importancia del hábitat de arroyo en predecir la presencia de especies de murciélagos. Registramos siete gremios; con similar número de gremios entre sitios de bosque y sitios de franjas de bosque ripario. En relación al hábitat de matriz, los hábitats de arroyo y borde en los sitios de franjas de bosque ripario fueron funcionalmente mas diversos, apoyando nuestra hipótesis sobre el potencial de valor de conservación de las franjas de bosque ripario. Los resultados de este estudio sugieren que el mantenimiento de las franjas de bosque ripario dentro de áreas abiertas para agricultura pueden ayudar a conservar la diversidad de murciélagos taxonómica y funcional. Este estudio también provee información básica para evaluar la eficacia del mantenimiento de estas características del paisaje para la mitigación de los impactos del desarrollo de la agricultura sobre la biodiversidad. Sin embargo, advertimos que las franjas de bosque riparios por si mismas no son suficientes para el mantenimiento de la biodiversidad; su valor depende del mantenimiento de extensas áreas de bosque cercanas.

**Palabras clave:** agricultura, Chiroptera, conservación, Perú, Phyllostomidae

Table A-1. Species registered during the sampling period by habitat and treatment. F = forest, FS = stream habitat in forest, RS = riparian forest strips in open areas cleared of forest. (BAerIns) background cluttered space-aerial-insectivores, (HAerIns) highly cluttered space-aerial-insectivores, (HCIns) highly cluttered space-gleaning-insectivores, (Car) highly cluttered space-gleaning-carnivores, (San) highly cluttered space-gleaning-sanguivores, (CanFru) highly cluttered space-gleaning-canopy-frugivores, (ShrFru) highly cluttered space-gleaning-shrub-frugivores, (Nec) highly cluttered space-gleaning-nectarivores, (Omn) highly cluttered space-gleaning-omnivores.

| Species | F | FS | | RS | | | Total | Trophic guild | Deposit code |
| --- | --- | --- | --- | --- | --- | --- | --- | --- | --- |
|  | Forest | Forest | Stream | Edge | Matrix | Stream |  |  |  |
| *Artibeus lituratus* | 18 | 10 | 11 | 5 | 2 | 7 | 53 | CanFru |  |
| *Artibeus obscurus* | 24 | 17 | 15 | 12 | 3 | 28 | 99 | CanFru |  |
| *Artibeus planirostris* | 40 | 54 | 35 | 7 | 4 | 31 | 171 | CanFru |  |
| *Carollia benkeithi* | 0 | 5 | 19 | 14 | 3 | 12 | 53 | ShrFru |  |
| *Carollia brevicauda* | 15 | 25 | 12 | 17 | 27 | 25 | 121 | ShrFru | CEBIOMAS0430 |
| *Carollia perspicillata* | 23 | 21 | 17 | 23 | 32 | 39 | 155 | ShrFru |  |
| *Chiroderma trinitatum* | 0 | 0 | 0 | 0 | 1 | 0 | 1 | CanFru |  |
| *Chiroderma villosum* | 0 | 0 | 1 | 0 | 1 | 0 | 2 | CanFru |  |
| *Chrotopterus auritus* | 1 | 0 | 0 | 0 | 0 | 0 | 1 | Car |  |
| *Dermanura anderseni* | 1 | 3 | 1 | 4 | 4 | 2 | 15 | CanFru | CEBIOMAS0433, CEBIOMAS0434 |
| *Dermanura* cf. *cinerea* | 0 | 0 | 0 | 0 | 1 | 1 | 2 | CanFru | CEBIOMAS0435 |
| *Dermanura glauca* | 0 | 1 | 0 | 0 | 0 | 0 | 1 | CanFru |  |
| *Dermanura gnoma* | 0 | 0 | 1 | 0 | 1 | 1 | 3 | CanFru |  |
| *Desmodus rotundus* | 7 | 10 | 6 | 14 | 2 | 20 | 59 | San |  |
| *Glossophaga soricina* | 1 | 0 | 0 | 0 | 4 | 0 | 5 | Nec |  |
| *Lionycteris spurelli* | 0 | 0 | 0 | 1 | 0 | 0 | 1 | Nec |  |
| *Lonchophylla thomasi* | 1 | 1 | 0 | 1 | 3 | 2 | 8 | Nec |  |
| *Lophostoma carrikeri* | 0 | 0 | 2 | 0 | 0 | 1 | 3 | HCIns |  |
| *Lophostoma silvicolum* | 1 | 1 | 2 | 1 | 2 | 4 | 11 | HCIns |  |
| *Mesophylla macconnelli* | 4 | 8 | 4 | 0 | 0 | 1 | 17 | ShrFru |  |
| *Micronycteris hirsuta* | 0 | 0 | 0 | 0 | 0 | 1 | 1 | HCIns |  |
| *Micronycteris megalotis* | 0 | 1 | 0 | 0 | 0 | 0 | 1 | HCIns |  |
| *Micronycteris minuta* | 0 | 0 | 0 | 0 | 1 | 2 | 3 | HCIns | CEBIOMAS0431 |
| *Mimon crenulatum* | 2 | 0 | 1 | 0 | 0 | 0 | 3 | HCIns |  |
| *Phylloderma stenops* | 0 | 1 | 0 | 0 | 0 | 1 | 2 | ShrFru |  |
| *Phyllostomus discolor* | 0 | 0 | 0 | 3 | 0 | 1 | 4 | Omn |  |
| *Phyllostomus elongatus* | 3 | 1 | 1 | 4 | 1 | 5 | 15 | Omn |  |
| *Phyllostomus hastatus* | 8 | 15 | 6 | 84 | 2 | 84 | 199 | Omn |  |
| *Platyrrhinus incarum* | 0 | 0 | 0 | 0 | 2 | 1 | 3 | CanFru |  |
| *Rhinophylla fischerae* | 0 | 1 | 3 | 2 | 1 | 0 | 7 | ShrFru |  |
| *Rhinophylla pumilio* | 46 | 18 | 10 | 6 | 5 | 9 | 94 | ShrFru |  |
| *Sturnira* cf. *luisi* | 0 | 0 | 1 | 0 | 0 | 2 | 3 | ShrFru | CEBIOMAS0429 |
| *Sturnira lilium* | 2 | 4 | 8 | 1 | 17 | 13 | 45 | ShrFru |  |
| *Sturnira magna* | 0 | 1 | 1 | 0 | 1 | 2 | 5 | ShrFru |  |
| *Sturnira tildae* | 0 | 1 | 1 | 0 | 5 | 10 | 17 | ShrFru |  |
| *Tonatia saurophila* | 2 | 3 | 0 | 2 | 1 | 1 | 9 | HCIns |  |
| *Trachops cirrhosus* | 0 | 5 | 0 | 0 | 0 | 1 | 6 | Car |  |
| *Trinycteris nicefori* | 1 | 1 | 0 | 0 | 0 | 1 | 3 | HCIns | CEBIOMAS0427 |
| *Uroderma bilobatum* | 3 | 2 | 2 | 3 | 4 | 1 | 15 | ShrFru |  |
| *Uroderma magnirostrum* | 1 | 0 | 0 | 2 | 0 | 0 | 3 | ShrFru |  |
| *Vampyressa thyone* | 1 | 3 | 0 | 0 | 0 | 0 | 4 | CanFru |  |
| *Vampyriscus bidens* | 11 | 1 | 0 | 0 | 0 | 0 | 12 | ShrFru |  |
| *Vampyriscus brockii* | 0 | 2 | 0 | 0 | 0 | 0 | 2 | CanFru |  |

Table A-2. Guild classification for all bat species (suborder Microchiroptera), following Kalko 1997 and Schnitzler & Kalko 1998, with a modification that separates highly cluttered space gleaning frugivores into shrub or canopy species.

Guild

Uncluttered space-aerial-insectivores

Background cluttered space-aerial-insectivores

Highly cluttered space-aerial-insectivores

Highly cluttered space-gleaning-insectivores

Highly cluttered space-gleaning-carnivores

Highly cluttered space-gleaning-piscivores

Highly cluttered space-gleaning-sanguivores

Highly cluttered space-gleaning-canopy-frugivores

Highly cluttered space-gleaning-shrub-frugivores

Highly cluttered space-gleaning-nectarivores

Highly cluttered space-gleaning-omnivores

References

Kalko, E. K. V. (1997). Diversity in tropical bats. In: Ulrich, H. (Ed.). *Tropical biodiversity and Systematics*. Zool. Forschungsinstitut und Museum Alexander Koenig, Bonn. Pp. 13-43.

Schnitzler, H. U., & Kalko, E. K. V. (1998). How echolocation bats fly and find food. In: Kunz, T. H., & Racey, P. A. (Eds.). *Bat Biology and Conservation*. Washington (DC): Smithsonian Institution Press. Pp. 183–196.

Table A-3. Number of individuals and species captured by habitat and by site. SE = standard error.

| **Treatment Site** | **Position** | **Habitat** | **Observed Species** | **Chao 1** | **Chao 1 - SE** | **Jackknife 1** | **Jackknife 1 - SE** | **Individuals captured** |
| --- | --- | --- | --- | --- | --- | --- | --- | --- |
| F1 | e | forest | 10 | 17.76 | 11.32 | 13.88 | 1.94 | 33 |
| F1 | m | forest | 12 | 14.59 | 3.40 | 15.89 | 1.95 | 36 |
| F1 | s | forest | 11 | 12.09 | 1.72 | 13.90 | 1.68 | 31 |
| F2 | e | forest | 12 | 24.22 | 16.76 | 16.89 | 2.19 | 44 |
| F2 | m | forest | 10 | 13.86 | 5.12 | 13.86 | 1.93 | 28 |
| F2 | s | forest | 11 | 23.21 | 16.76 | 15.89 | 2.18 | 44 |
| FS1 | e | forest | 10 | 16.05 | 7.32 | 14.84 | 2.16 | 31 |
| FS1 | m | forest | 17 | 29.10 | 12.99 | 23.91 | 2.61 | 80 |
| FS1 | s | stream | 19 | 22.10 | 3.63 | 23.96 | 2.22 | 122 |
| FS2 | e | forest | 17 | 29.05 | 12.95 | 23.89 | 2.60 | 61 |
| FS2 | m | forest | 16 | 19.52 | 3.77 | 21.86 | 2.39 | 44 |
| FS2 | s | stream | 15 | 22.95 | 8.07 | 21.81 | 2.58 | 38 |
| RS1 | e | edge | 16 | 17.12 | 1.76 | 18.98 | 1.72 | 132 |
| RS1 | m | matrix | 21 | 35.94 | 12.32 | 33186 | 3.27 | 81 |
| RS1 | s | stream | 26 | 109.96 | 96.45 | 38.92 | 3.58 | 156 |
| RS2 | e | edge | 15 | 16.48 | 2.26 | 17.96 | 1.70 | 74 |
| RS2 | m | matrix | 14 | 26.25 | 16.80 | 18.90 | 2.19 | 49 |
| RS2 | s | stream | 21 | 65.70 | 29.96 | 30.93 | 3.14 | 153 |

Table A-4. Species loadings for the generation of a non-multidimensional scaling analysis (NMDS) using the sites by treatment data.

| Species | Axis 1 | Axis 2 |
| --- | --- | --- |
| *Artibeus lituratus* | -0.051 | 0.150 |
| *Artibeus obscurus* | 0.023 | -0.010 |
| *Artibeus planirostris* | -0.013 | -0.015 |
| *Carollia benkeithi* | 0.054 | 0.000 |
| *Carollia brevicauda* | 0.013 | -0.009 |
| *Carollia perspicillata* | 0.043 | 0.017 |
| *Chiroderma trinitatum* | 0.223 | 0.000 |
| *Chiroderma villosum* | 0.223 | 0.000 |
| *Chrotopterus auritus* | -0.149 | 0.360 |
| *Dermanura anderseni* | 0.020 | 0.058 |
| *Dermanura* cf*. cinerea* | 0.223 | 0.000 |
| *Dermanura glauca* | 0.223 | 0.000 |
| *Dermanura gnoma* | 0.223 | 0.000 |
| *Desmodus rotundus* | 0.059 | 0.024 |
| *Glossophaga soricina* | 0.060 | 0.158 |
| *Lionycteris spurelli* | 0.223 | 0.000 |
| *Lonchophylla thomasi* | 0.005 | 0.117 |
| *Lophostoma carrikeri* | 0.223 | 0.000 |
| *Lophostoma silvicolum* | 0.142 | -0.078 |
| *Mesophylla macconnelli* | -0.067 | 0.156 |
| *Micronycteris hirsuta* | 0.223 | 0.000 |
| *Micronycteris megalotis* | 0.223 | 0.000 |
| *Micronycteris minuta* | 0.223 | 0.000 |
| *Mimon crenulatum* | -0.202 | -0.275 |
| *Phylloderma stenops* | -0.176 | 0.000 |
| *Phyllostomus discolor* | 0.223 | 0.000 |
| *Phyllostomus elongatus* | -0.028 | -0.036 |
| *Phyllostomus hastatus* | 0.101 | 0.004 |
| *Platyrrhinus incarum* | 0.223 | 0.000 |
| *Rhinophylla fischerae* | 0.107 | 0.000 |
| *Rhinophylla pumilio* | -0.102 | -0.029 |
| *Sturnira* cf*. luisi* | -0.049 | 0.000 |
| *Sturnira lilium* | 0.126 | 0.004 |
| *Sturnira magna* | 0.059 | 0.000 |
| *Sturnira tildae* | 0.165 | 0.000 |
| *Tonatia saurophila* | -0.134 | 0.015 |
| *Trachops cirrhosus* | -0.231 | 0.000 |
| *Trinycteris nicefori* | 0.011 | -0.205 |
| *Uroderma bilobatum* | 0.031 | 0.061 |
| *Uroderma magnirostrum* | -0.010 | -0.225 |
| *Vampyressa thyone* | -0.293 | -0.126 |
| *Vampyriscus bidens* | -0.135 | -0.197 |
| *Vampyriscus brockii* | -0.370 | 0.000 |

Table A-5. Species loadings for the generation of a non-multidimensional scaling analysis (NMDS) using the habitat data.

| Species | Axis 1 | Axis 2 |
| --- | --- | --- |
| *Artibeus lituratus* | -0.156 | 0.014 |
| *Artibeus obscurus* | -0.081 | -0.017 |
| *Artibeus planirostris* | -0.105 | -0.037 |
| *Carollia benkeithi* | 0.141 | -0.03 |
| *Carollia brevicauda* | 0.074 | -0.011 |
| *Carollia perspicillata* | 0.046 | 0.017 |
| *Chiroderma trinitatum* | 0.402 | 0.132 |
| *Chiroderma villosum* | 0.236 | 0.095 |
| *Chrotopterus auritus* | -0.478 | -0.014 |
| *Dermanura anderseni* | 0.21 | -0.112 |
| *Dermanura* cf*. cinerea* | 0.342 | -0.072 |
| *Dermanura glauca* | -0.2 | -0.417 |
| *Dermanura gnoma* | 0.203 | 0.083 |
| *Desmodus rotundus* | 0.006 | -0.02 |
| *Glossophaga soricina* | 0.135 | -0.075 |
| *Lionycteris spurelli* | 0.227 | -0.066 |
| *Lonchophylla thomasi* | 0.031 | 0.063 |
| *Lophostoma carrikeri* | 0.103 | 0.121 |
| *Lophostoma silvicolum* | 0.091 | 0.08 |
| *Mesophylla macconnelli* | -0.124 | -0.115 |
| *Micronycteris hirsuta* | 0.21 | 0.283 |
| *Micronycteris megalotis* | -0.112 | -0.048 |
| *Micronycteris minuta* | 0.286 | 0.223 |
| *Mimon crenulatum* | -0.136 | -0.096 |
| *Phylloderma stenops* | 0.194 | -0.027 |
| *Phyllostomus discolor* | 0.101 | 0.27 |
| *Phyllostomus elongatus* | -0.046 | 0.12 |
| *Phyllostomus hastatus* | 0.066 | 0.191 |
| *Platyrrhinus incarum* | 0.349 | 0.173 |
| *Rhinophylla fischerae* | 0.131 | -0.073 |
| *Rhinophylla pumilio* | -0.131 | -0.016 |
| *Sturnira* cf*. luisi* | 0.13 | -0.106 |
| *Sturnira lilium* | 0.138 | -0.022 |
| *Sturnira magna* | 0.165 | 0.093 |
| *Sturnira tildae* | 0.239 | 0.114 |
| *Tonatia saurophila* | 0.058 | -0.002 |
| *Trachops cirrhosus* | -0.089 | 0.054 |
| *Trinycteris nicefori* | -0.076 | -0.055 |
| *Uroderma bilobatum* | 0.064 | -0.168 |
| *Uroderma magnirostrum* | -0.128 | 0.297 |
| *Vampyressa thyone* | -0.107 | 0.206 |
| *Vampyriscus bidens* | -0.317 | 0.182 |
| *Vampyriscus brockii* | 0.068 | 0.05 |

Figure A-1. Individual-based rarefaction curves for the bat species at six sampling sites. Doted and dashed lines represent 95% confidence intervals. Treatments: F = forest, FS = stream habitat in forest, RS = riparian forest strips in open areas cleared of forest.

A
B

C

Figure A-2. Percentage of capture rate by habitat for bat species. A) FS treatment, B) RS1 and C) RS2. FS = stream habitat in forest, RS = riparian forest strips in open areas cleared of forest.

Figure A-3. Rank abundance curves for all sites. F = forest, FS = stream habitat in forest, RS = riparian forest strips in open areas cleared of forest.
